# Visual Participatory Analysis: A Qualitative Method for Engaging Participants in Interpreting the Results of Randomized Controlled Trials of Health Interventions

**DOI:** 10.1177/1558689820914806

**Published:** 2020-04-13

**Authors:** Jenevieve Mannell, Katy Davis, Kohenour Akter, Hannah Jennings, Joanna Morrison, Abul Kuddus, Edward Fottrell

**Affiliations:** 1 University College London, UK; 2 University of Leeds, UK; 3 Diabetic Association of Bangladesh, Dhaka, Bangladesh; 4 University of York, UK

**Keywords:** participatory analysis, randomized controlled trials, diabetes, Bangladesh

## Abstract

This article contributes to the field of mixed methods by introducing a new method for eliciting participant perspectives of the quantitative results of randomized controlled trials. Participants are rarely asked to interpret trial results, obscuring potentially valuable information about why a trial either succeeds or fails. We introduce a unique method called visual participatory analysis and discuss the insights gained in its use as part of a trial to prevent risk and reduce the prevalence of diabetes in Bangladesh. Findings highlight benefits such as elucidating contextualized explanations for null results and identifying causal mechanisms, as well as challenges around communicating randomized controlled trial methodologies to lay audiences. We conclude that visual participatory analysis is a valuable method to use after a trial.

Cluster randomized controlled trial (RCT) designs have been widely used to evaluate community-based interventions addressing health issues from child mortality to gender-based violence (Ellsberg et al., 2015; Prost et al., 2013). However, interpreting the quantitative results of these trials largely remains a scholarly endeavor carried out by research teams with sophisticated academic skills. This results in interpretations that are often read and understood by highly trained professionals, limiting the potential for wider dissemination of knowledge into practice (Brownson et al., 2017) and public engagements with science (Snow & Dibner, 2016). In some cases, the absence of adequate qualitative research in developing and implementing quantitative trials may also contribute to a poor interpretation of trial results based on a misinterpretation of the local context (Elford et al., 2001; Wight et al., 2002), or a lack of knowledge about what actually occurred during an intervention (Pope & Mays, 1995).

Past oversight and potential errors have resulted in new approaches and guidelines for RCTs that explicitly promote the use of mixed methods as part of intervention evaluation (Davis et al., 2019; Drabble & O’Cathain, 2015). While evaluations that give equal weight to both qualitative and quantitative data have been slow to come about in practice (Lewin et al., 2009; Rapport et al., 2013), current guidelines recommend the use of qualitative process evaluations alongside quantitative RCTs as a means of gathering information about how the intervention and the trial were implemented (Craig et al., 2008; Craig et al., 2013). It is recommended that process evaluation findings be used in interpreting quantitative RCT results to expose potential reasons for deviations from the trial protocol during implementation, clarify causal mechanisms of the intervention, or identify contextual factors that may have had an influence on outcomes (Moore et al., 2015). Despite these recommendations, however, qualitative research conducted alongside trials is rarely discussed in the interpretation of published trial results (Drabble et al., 2014; O’Cathain et al., 2014) and often has major methodological shortcomings (Lewin et al., 2009).

The purpose of this article is to introduce a unique mixed methods approach to evaluation research, one which relies on the participation of those targeted by the intervention in the interpretation of the quantitative results. The participation of groups or individuals targeted by interventions as part of the research process has been widely supported by qualitative researchers for decades, in some cases, contributing to the development of entirely new methodological approaches, such as community-based participatory research (Minkler & Wallerstein, 2008), participatory rural appraisal (Chambers, 1994), and participatory action research (Lewin, 1946). These approaches belong to a well-established body of methodological literature about engaging participants in the research process. Participatory techniques, including photovoice, mapping, issue prioritization, and time analysis tools, are often used as a means of collecting valuable data with illiterate populations, children, or marginalized communities (Skovdal & Cornish, 2015; Wang & Burris, 1997). The use of peer researchers as data collectors has also been widely used within health as a means of overcoming power dynamics that may exist between researchers and participants (MacLellan et al., 2017). Others have developed new approaches to including research participants in the analysis process, such as involving stakeholders in team-based analysis (Flicker & Nixon, 2015) and using emoticons to evaluate programs with people living with disabilities (Post et al., 2016). The perceived advantages of the participation of targeted communities in research processes include the potential to address the needs of communities that have been previously marginalized by research (Smith, 1999), the ability to gain diverse perspectives from local stakeholders (Flicker & Nixon, 2015), and the deepening of analytical insights through local engagement (Karim et al., 2016).

Participatory research methods have rarely been used as part of RCTs, with a few notable exceptions (Davis et al., 2019). One example of this is “Broad Brush Surveys” that include participatory community-based activities, and have been suggested as a comprehensive technique for collecting rich case studies about the local context to inform the design of a trial (Bond et al., 2019). Communities have also been involved in the design of trials using techniques such as public randomization procedures, and the co-design of research tools in collaboration with research participants (Flicker et al., 2010). However, the vast majority of participatory approaches that have been used within trials are part of the intervention rather than its evaluation, and involving participants in the analysis process is virtually unheard of in trials.

This article draws on the participatory approach to qualitative health research to develop knowledge about the process and utility of using participatory research methods as part of a mixed methods analysis of trial data. It does this by describing how an alternative approach to data interpretation that involves the participation of targeted communities in the interpretive process—visual participatory analysis (VPA)—was used as part of the DMagic trial in Bangladesh. The article begins with a description of the DMagic trial to provide a contextual background to the intervention evaluation that led to the development of the VPA method. We then describe the development of the methodology and how the data were collected and analyzed using this method. The findings summarize the results of our analysis of the VPA data followed by a discussion of how these findings contribute to both a better understanding of the DMagic intervention and to the mixed methods literature.

## The DMagic Trial

The Diabetes Mellitus Action through Groups or Mobile Information for better Control (DMagic) trial was a three-arm cluster RCT conducted in 96 rural villages (clusters) in Faridpur district, Bangladesh, covering a population of approximately 125,000 (Fottrell et al., 2016). Based on public randomization process, 32 villages were allocated to receive a participatory learning and action (PLA) group intervention, 32 were allocated to receive mHealth health promotion voice messages delivered to mobile phones, and 32 were allocated to usual care, which in this context is care seeking in government or private facilities often associated with out-of-pocket payments, and limited preventative public health campaigning.

The PLA intervention consisted of monthly group meetings whereby community members were guided through a process of problem identification and prioritization, strategy planning to overcome identified problems, implementation of strategies, and evaluation. Groups were facilitated by local men and women who were recruited by the project team and given training on group facilitation and key concepts in diabetes prevention and control. Separate men and women’s groups were established to ensure women felt comfortable speaking freely given the highly gendered context of Bangladesh, but there were no restrictions on participation in the groups. A total of 122 groups were established across 32 villages and an average of 27 people participated in each group on a monthly basis over 18 months. Group strategies varied but often involved community awareness raising, exercising in groups, coordination of blood sugar testing and small income-generating and kitchen-gardening activities.

The mHealth intervention consisted of short twice-weekly voice messages on the signs, symptoms, prevention, and care for diabetes sent to participants’ mobile phones over a period of 14 months. Anyone residing in the 32 mHealth intervention villages with a mobile phone was eligible to receive the messages on registration with the intervention team. A range of message formats was used, including information giving, dramas, and songs. All content was based on formative research and behavior change theories and was reviewed by medical experts (Jennings et al., 2019; Morrison et al., 2019).

Trial impact was measured through sample surveys of individuals living within the study villages and has been reported elsewhere (Fottrell et al., 2019). Primary outcomes were the following: (a) population prevalence of diabetes and intermediate hyperglycemia (defined as impaired fasting glucose and/or impaired glucose tolerance) among adults aged 30 years and older and (b) 2-year cumulative incidence of diabetes among adults 30 years and older identified with intermediate hyperglycemia before intervention. Large, significant reductions were observed in the prevalence of type 2 diabetes and intermediate hyperglycemia in the PLA intervention arm compared with control (adjusted odds ratio [95% confidence interval], 0.36 [0.27, 0.48]), and in the 2-year cumulative incidence of type 2 diabetes among an intermediate hyperglycemic cohort in the PLA arm compared with control, 0.39 [0.24, 0.65]. No differences in primary outcomes were observed between the mHealth arm and control. Secondary outcomes included measures of knowledge and awareness of diabetes which improved significantly in both intervention arms relative to control, although the impact was consistently higher in the PLA intervention arm. Additional secondary outcome measures of blood pressure, obesity, quality of life, care seeking, physical activity, and fruit and vegetable consumption did not differ significantly between any of the trial arms.

## Method

The main research question guiding this substudy of the DMagic trial was the following: “How do community members participating in all three arms explain the trial’s main quantitative results?” Once the preliminary results of the trial were known to the research team, this was supplemented by the following subquestion: “How do community members explain the impact of the PLA group intervention and the lack of observed effect of the mHealth intervention on diabetes outcomes?” We developed VPA as a method suited to answering these research questions. The study was included under the ethical approvals obtained for the DMagic trial (UCL ethics approval 4766/002; Diabetic Association of Bangladesh ethics approval BADAS-ERC/EC/t5100246)), and provisions for feeding back the trial results to participants. The DMagic trial is registered with the ISRCTN registry, number ISRCTN41083256.

VPA draws on an interpretivist approach to qualitative research that aims to explore the meanings individuals hold for social objects or behaviors and the ways in which these are socially constructed through interpersonal interaction and modes of communication (Schwandt, 1998). From this perspective, involving research participants in the analysis of trial data offers a means of accessing the meaning that individuals attribute to their behaviors as reflected in the data. As such, VPA provides a means of involving communities in the analysis of research results drawing on an interpretivist interest in the co-construction of research through open communication and reflection between the research team and the participants.

### Initial Tool Design—VPA

In designing VPA for the DMagic trial, the project team first reviewed the quantitative results and selected a list of outcomes according to: their local relevance and potential to be understood by the participants (e.g., the exclusion of purely medical outcomes that had little relevance to people’s everyday lives), and their lack of a clear explanation for why an outcome was achieved at the level or in the direction it was achieved, which would benefit from participant input (see Table 1).

**Table 1.** Results From DMagic Selected by the Research Team for Visual Participatory Analysis.

Intervention		Outcome of interest	Description of how the results should be visually represented (e.g., Results)
**Community groups**	1	Self-awareness of diabetic status (comparing self-reports with blood glucose levels)	After the community groups, five times as many people were aware of whether they have diabetes or not than before the community groups.
	2	Awareness of the symptoms of diabetes	After the community groups, 24 times as many people were aware of the symptoms of diabetes than before the community groups.
	3	Utilization of diabetic services	Care seeking from diabetic services was already good, but after the community groups, the same number of people used diabetic health services.
	4	Smoking prevalence	After the community groups the same number of people smoked as before
	5	Prevalence of overweight and obesity	After the community groups there was no change in obesity.
	6	Physical activity graded according to the intensity and duration of work (heavy, moderate, mild, and sedentary, based on an equivalent walk of >90 min, 60-90 min, 30-59 min, and <30 min/24 hours, respectively)	After the community groups people did about 60 minutes more exercise a week.
	7	The combined prevalence of intermediate hyperglycemia (i.e., impaired fasting glucose or impaired glucose tolerance) and type 2 diabetes mellitus among adults aged 30 years or older	In the villages that had community groups, the number of people with diabetes had decreased by half.
**mH**ealth	8	Self-awareness of diabetic status	Awareness was improved.
	9	The combined prevalence of intermediate hyperglycemia (i.e., impaired fasting glucose or impaired glucose tolerance) and type 2 diabetes mellitus among adults aged 30 years or older	There was no change in prevalence.
	10	Quality of life	Reported quality of life improved after the mHealth intervention.

We then worked with a local artist in Bangladesh to represent the selected intervention outcomes as drawings, with one drawing representing each outcome of interest. In some cases, the trial outcomes differed substantially from the outcomes represented in the drawings to address challenges in communicating complex outcome measures (e.g., physical activity for the trial was measured as the percentage of individuals doing at least 150 minutes of physical activity per week, which was difficult to represent visually). In other cases, the drawings provided a means of investigating interesting results from the trial that had not been specified as primary or secondary trial outcomes (e.g., smoking behavior). Working with a local artist helped to ensure that the drawings represented local dress, food, and environment of the Faridpur region of Bangladesh, where the trial took place. The project team reviewed the initial drawings to ensure the results were accurately represented and understandable, and small modifications were made by the local artist.

**Figure 1.**
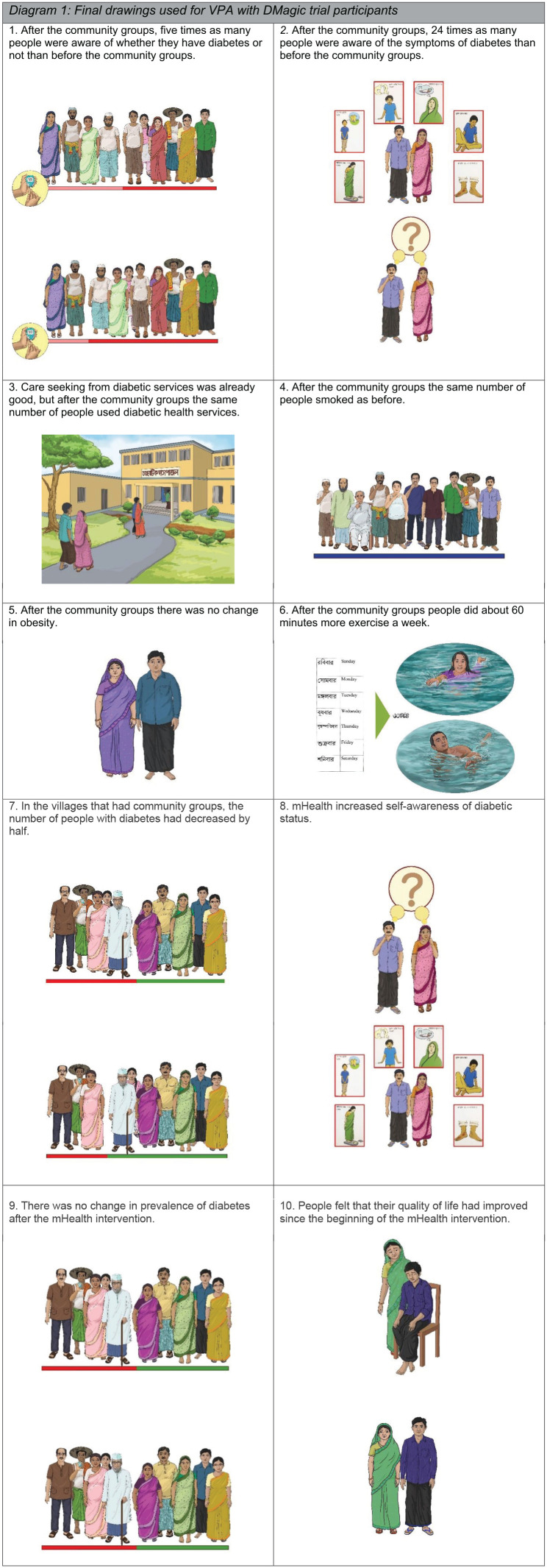
Final drawings.

Once the drawings were finalized, the project team field tested the drawings with four community advisory committees (CACs)—groups of community representatives from the study areas involved in the DMagic trial. This involved sharing the drawings with all four CACs involved in the trial and facilitating a group discussion following a topic guide that asked what participants thought the drawings meant; the use of colors, and how effective these were at communicating the message; the use of a timeline bar in several drawings and whether this had meaning; and the appropriateness of how people in the drawings were represented (e.g., clothing, environment, gender). The four CAC group discussions were attended by 6 to 10 community representatives and lasted 90 to 120 minutes each. Three members of the research team (KA, HJ, and TN) attended the meetings and took turns facilitating the meetings and taking notes. Following each meeting, they shared notes and discussed the feedback received during the meeting. Their feedback was compiled and shared with the broader project team. The project team then used the information gathered during the CAC discussions to inform a new round of changes to the drawings in collaboration with the local artist (see Figure 1).

### Participant Selection and Recruitment

The project team selected two villages from each of the three trial arms to recruit participants to participate in the VPA (mHealth intervention, PLA intervention, and control). We excluded villages that had been approached for other process evaluation research to maximize variety of the data collected as part of the trial and not to overburden any single community. Field coordinators who had supervised group facilitators in the DMagic trial approached participants to attend VPA group discussions in each of the communities. We purposively selected these participants to obtain a balance between those who had been involved directly in the intervention (e.g., had received mHealth messages or participated in the PLA intervention), and those who had not, and to achieve a gender balance. Verbal consent was taken from participants for their involvement in the discussions.

### Data Collection

The project team carried out two VPA group discussions (one with men and one with women) in each of the three trial arms. Approximately, seven to nine individuals participated in each of the group discussions, and on average the discussions lasted between 90 and 120 minutes. The third author (KA) facilitated these group discussions, and the second (KD) and fifth (JMo) authors observed. During the VPA group discussions, KA first presented the artist’s drawings representing the intervention and asked participants their understanding of the intervention and the research process. Next, KA presented the drawings representing the results of the trial in a series of picture pairs (each pair representing an outcome before and after the intervention). First, KA revealed the preintervention drawing and asked participants what they thought the drawing showed. She then asked them how they thought this outcome would change after the intervention through a show of hands (to keep discussion short). Second, KA revealed the postintervention drawing and again asked participants what they thought it showed. Once they understood the represented change (or lack of), KA asked participants what they thought was the cause of change (or lack of). The project team iteratively developed a series of questions about specific results to test theories that emerged in the data, which were then incorporated into the topic guide. VPA group discussions were recorded and later transcribed into English for analysis.

### Data Analysis

The facilitator and observers took detailed notes during the VPA group discussions, including analytical insights by the research team as well as reflections on the positionality of team members and its potential implications for the discussions that took place (e.g., the influence of foreign observers on group discussions). These field notes were later entered into NVIVO for analysis together with the transcripts. The first author (JM) performed a thematic analysis of the data by first reading through the transcripts/field notes, and making memos of initial thoughts and emergent ideas about potential themes. Following this initial process, JM completed a detailed thematic analysis in NVIVO to identify both deductive and inductive themes that responded directly to the research questions (Attride-Stirling, 2001). This involved first creating a coding framework from observational field notes, memos, a subset of three group discussions (one from each arm), and associating codes with the transcripts in NVIVO. Codes that appeared multiple times across different transcripts were organized into basic themes and those with only a single instance were discarded. This process also allowed for inductive coding, whereby new themes were iteratively identified during analysis. After coding all transcripts, the basic themes were grouped into organizing themes, which were then analyzed for their broader analytical potential to answer the research question. At this stage, the preliminary thematic analysis was reviewed by those involved in facilitating and observing the group discussions to ensure the findings accurately reflected their experiences from the field. The insights of everyone involved in the data collection and analysis have been used in developing the final analysis of the data and the grouping of organizing themes into the global themes presented in this article.

## Findings

The findings presented in this article relate specifically to the use of the VPA method, its advantages and challenges in providing participant perspectives on the quantitative trial data. Advantages of using the VPA method explored in these findings include the method’s ability to provide a rich contextual explanation of null results on care-seeking behaviors and to explore participant perspectives on what contributed to the success of the PLA intervention. However, the findings also reveal a challenge of the VPA method related to effectively communicating RCT methodologies to participants, which contributed to feelings by participants that the data presented did not reflect their experience of the intervention. Each of these findings are described in detail.

### Participants’ Explanations of Null Results on Care-Seeking Behaviors

Image No. 3 in Figure 1 presents findings that there had been no change in care-seeking behaviors as a result of the intervention. This image caused considerable debate among participants about why these changes had not taken place. The main explanation provided for the lack of change across the groups was that people were managing their diabetes better and therefore did not need to go to the doctor:

Participant 1: It didn’t increase because people have become more aware now, they are maintaining their routine, doing their tests, living a disciplined life, so as a result people would be less afflicted with diabetes. That’s why. (VPA group discussion with men in PLA intervention area)

Similar explanations were also discussed by participants in control areas of the trial:

Participant 1: People are conscious now, so the number did not increase. The number of diabetes patients decreases day by day.

Participant 2: He means that people know a lot about diabetes nowadays. So, they avoid many of the things that can cause diabetes.

Participant 3: People are controlling their habits and maintaining a safe life. (VPA group discussion with men in control area)

This explanation was heard in all arms of the intervention and by both men and women. It is a reasoned explanation for why the finding around care-seeking behavior showed no change as a result of the intervention. If the intervention was indeed successful in terms of reducing preventative behaviors then care-seeking may have become less necessary. In this way, the input of the participants provided additional evidence to support the interpretation of null results in the quantitative data.

### Participant’s Perspectives on What Contributed to the Success of the PLA Intervention

Before participants were told about the success of the PLA intervention and lack of observed effect from the mHealth intervention, participants in groups discussions across all three arms consistently predicted this result. Broadly, participants felt that facilitated group discussions were far more effective in developing their knowledge about risk factors for diabetes and prevention-related behaviors and in enabling them to change existing practices than mHealth mobile voice messages.

During the group discussions, participants gave several reasons for this. First, group conversations with a knowledgeable facilitator were perceived to provide an opportunity for individuals to ask questions about diabetes:

Participant 1: Well, we understand better when face to face.

Participant 2: We can understand and we can ask questions. (VPA group discussion with men in PLA intervention area)

Participants also felt that the opportunity for face-to-face interaction and the use of visual and participatory tools helped improve their understanding. Participants described how the group discussions were a means of turning knowledge about diabetes into conscious action and therefore changes in behavior:

Participant 1: Reality is different. Here we are listening. But if we do not take actions accordingly, then it will not be possible to implement.

Participant 2: [Mobile messages] are all about listening and [group discussions] are about watching.

Participant 1: Right. Listening and Watching. We are more likely to understand instructions when we watch.

Participant 3: We have to take actions accordingly. There is no point learning if you don’t act on what you have learned.

Participant 1: There is a difference between watching a television and listening to a radio. Here the difference is similar. We can listen to our mobile phones, but the message can only be heard. [Group discussions] can be watched. Naturally, if we watch something, it is easy to understand and can be easily remembered. Here, [in the group], we can watch the things practically. (VPA group discussion with men in mHealth intervention area)

Participants also shared quite complex explanations for how group discussion brings about broader social change across an entire community, and helps change behaviors beyond just those individuals participating in PLA intervention activities. Participants discussed the potential for group discussions to disseminate the messages beyond the intervention group and outward into the wider community through sharing with friends and family, and through informal social conversations:

Participant 6: This has happened because of our interconnection. It is like this: *didi* [sister] attended a meeting and she met with another *didi* later. They talked to each other. That’s how these things came up. They talked about it. Then that *didi* later chatted with another and thus awareness of diabetes spread. Everyone came to know about diabetes. (VPA group discussion with women in PLA intervention area)

This process of participating in the group activities and then sharing the message with others in the community was perceived to be a major advantage of the PLA group discussions over the mHealth intervention. When asked why people were more likely to share what they had learned in a group versus through mobile messages, participants said that group discussions were able to build trust in messaging that the mobile messages could not:

Facilitator: Why is [change] not possible by sending voice messages over mobile phones?

Participant 5: They [community members] only listened over mobile phones. They do not see who is talking. That is why they do not take it seriously. In meetings people can see each other. So they take it seriously and followed all the rules.

Participant 7: Talking face to face carries a value. You came here and spent a long time with us. You asked a lot of questions and got the answers. These things carry a value. It is not possible over phone. (VPA group discussion with women in a PLA intervention area)

This highlights several advantages of group-focused PLA activities over mHealth messaging according to the perspective of participants in both interventions. Participants felt that behavioral changes were much more likely to result from group activities because of the ability to assist with memory and understanding, but also in establishing the trust of participants in the messages being disseminated. Moreover, the public display of a meeting within a community setting contributed to the further dissemination of this trusted message through informal social conversations with others in the community who had heard about the meeting, but had not been able to attend.

### The Data Presented Did Not Reflect Participants’ Experience of the Intervention

Across all three arms of the intervention, participants contested the accuracy of the data presented and felt that some of the images were incorrect. For example, one participant disagreed with the number of people shown in Image No. 3 in Figure 1 about the number of people attending diabetes services:

Participant 1: Though, I think the number of people going to the hospitals has increased.

Participant 2: That’s what you think. But it is not really showing here.

Participant 3: The number of patients that we used to see before, have multiplied at least 3 times that now. (VPA group discussion with men in PLA intervention area)

The reasons given for incorrect data were wide reaching and included people lying (e.g., about not quitting smoking), the right data not being collected at the right time, people losing weight, changing behavior after post-intervention survey data were collected, or the data being collected from people who did not change behaviors. Overall, these objections by participants to the data arose primarily because participants had difficulties in reconciling the data with their own experiences of the intervention:

Facilitator: The number of people who were previously obese is still the same even after the meetings.

Participant 1: No, it isn’t true. The same number of people aren’t still obese today. It may be in the picture, but not in the original reality.

Facilitator: Not in the original reality. Why do you think this is, uncle?

Participant 1: We walk around spreading the messages to everyone, to make them aware. Everyone is becoming more aware. (VPA group discussion with men in a PLA intervention area)

The idea that data collected at a population level could be different from their own experience of the intervention did not seem possible to participants:

Facilitator: Alright. We found that the number of smokers remained the same before and after the meetings.

Participant 5: This is not true. Because those who attended meeting they have changed. I said that the persons in the picture did not attend the meeting. Those who attended meetings have changed themselves. We saw that in our village. (VPA group discussion with women in a PLA intervention area)

While some participants thought that this could be because people had lied to the survey team (e.g., around smoking behaviors), many participants explained this discord between their experience and the data as a problem with how the data were collected:

Participant 3: That’s not the fact. People have become more aware, it is true. You have not taken the data of the things we did. The things we, the people who attended meetings did, you have not taken the data of those people.

Participant 5: Everyone’s data is not included. People who learned [about diabetes], did change. They followed the rules. (VPA group discussion with women in a PLA intervention area)

This demonstrates one of the challenges of presenting population-level data obtained through surveys to small community groups, and the misunderstandings that can result among participants when individual experience contradicts research findings.

## Limitations

The VPA method encountered several challenges in presenting the results of the DMagic trial to participants in Bangladesh. In particular, participants struggled to understand trial results that showed a nonsignificant change in behavior, particularly if this contradicted their own changes in behavior or that of other community members. Their response in these situations was often that they had changed their behavior and that the problem must therefore be with the data or the way the evaluation was carried out. This kind of reaction may be counterproductive to the aims of an intervention by creating suspicion of researchers and evaluation processes in general. While this could potentially be avoided by reporting only positive results, this would also limit the potential benefits of the method. Another option would be to present individual community results that did show a change alongside population results that may not be statistically significant. This would allow facilitators to discuss the changes that did happen at a local level versus the broader trial results. However, such an option would also need to be carefully balanced against potential ethical challenges raised by stigmatized outcomes in communities where individuals could potentially be identified by sharing local level results (e.g., domestic violence or neonatal deaths).

The use of the VPA method in the DMagic trial was not very participatory in practice due to the need for a facilitator to explain the drawings and their meaning to the participating group, which was often done in a classroom style delivery. This may have limited the depth of data obtained and the extent to which the data reflect a broad range of potential interpretations and perceptions of the trial. However, this could easily be overcome by using multiple hand-held pictures and smaller group discussions observed by a facilitator followed by group feedback. If we had had more time to develop the VPA method, it would have also be advantageous to involve communities in the development of the drawings from the very beginning to ensure that they accurately reflected local perceptions of the intervention, social processes, and context.

In villages belonging to the control arm of the DMagic trial, participants in group discussions were largely motivated by trying to convince the facilitator to give them the intervention. In addition, participants that had no experience of the actual intervention found it more difficult to discuss the ways in which the intervention had been effective. The data from these areas were therefore often biased toward the possibility for behavioral changes to happen, rather than an authentic engagement with the trial results.

From a research team perspective, the process of developing visual representations of statistical results from the trial was extremely challenging and involved making compromises in the accuracy of the drawings. However, this limitation is the broader effect of the challenges that face researchers in translating their results into simple easy-to-understand language for public dissemination. Compromises will need to be made, and as a research team we decided that the accuracy of the results would need to come second to the clarity of the message.

## Discussion

The VPA method provided a valuable means of collecting data to inform the interpretation of quantitative trial results. Our findings point to several new pieces of data that came out of a post-trial discussion with participants that we would not have otherwise been fully understood from the process evaluation data or the quantitative results alone. Moreover, the data produced by this method provided a valuable means of triangulating the process evaluation data, contributing to the robustness of the results and understandings of the intervention overall.

### Perceived Benefits of Group Interventions for Message Dissemination

Our main finding was the response to our subquestion arising from the quantitative results around why the PLA intervention had achieved statistically significant results while the mHealth intervention had not. As summarized in the findings, participants spoke at length about the benefits of group interventions and the ways in which they would participate in a group activity and then tell their families, neighbors, and friends about what had taken place. However, people were also selective about the messages they shared with others, and perceived messages delivered in group discussions to be inherently more valuable and trustworthy than those delivered by mobile messages. This resonates with findings from the qualitative process evaluation of the DMagic trial that the group discussions gave confidence to members of the community who were illiterate and that their credibility was heightened with others in their community if they had attended a group meeting (Morrison et al., 2019). This points to the role of group and community interactions in the way individuals perceive the value of their own individual actions and behaviors (Howarth et al., 2004). Participants valued the group discussions because of the face-to-face interactions, time spent by the facilitators, and opportunities for discussion. For participants, the ability of the PLA intervention to spread messages about diabetes beyond the group that had participated directly in the discussions was rarely about peer pressure to conform. Participants saw value in diabetes messages that were delivered at a group level and were therefore more likely to spread these messages to others in their community.

This is consistent with the theoretical literature on the importance of shifting social norms as part of behavior change interventions rather than focusing only on individual attitudes or beliefs (Bandura, 2004). Other studies of diabetes and obesity interventions have presented similar findings. Group norms have been shown to shift eating behaviors and norms-based interventions recommended to promote healthy eating (Nook & Zaki, 2015). Studies of obesity point to the importance of social influence and social networks on eating behaviors (Hammond, 2010). Social norms about appropriate weight and body type shift and change over time and in different social locations, pointing to their potential to be changed at a social rather than individual level (Burke et al., 2010). The findings from this study support evidence that interventions are more effective at changing behavior when they challenge social norms, and that participants themselves support such conclusions when asked as part of VPA methods. Participants have sophisticated understandings of community dynamics and how these interact with changes in behavior, which can be used to inform interventions and their evaluation.

### Changes in Behavior Influenced Care Seeking

Another finding that arose from the VPA method included participants’ explanation of why we did not see a change in care-seeking behavior as part of the trial. As summarized in the findings, participants described how changes in their behavior in managing diabetes through healthy eating and increased levels of physical activity reduced their need to go to the doctor or hospital. Again, the understanding gained through the VPA method was integral to explaining this null result from a participant perspective. It also provides sophisticated insights from a participant perspective of the role that participatory behavior change interventions can play in influencing care-seeking behaviors.

This finding offers a contribution to the literature that is specifically gained through engaging participants in a discussion of the trial results—the analysis component of the VPA method. By asking participants about their reflections on the results, we are stepping away from a standard process evaluation. While process evaluations may engage participants in discussions about what they thought about an intervention, VPA focuses on sharing the actual results of the trial with participants to gain their direct input into the analysis process. This provides insight into how results are interpreted by the participants themselves, removing the potential misinterpretations that may occur when research teams are trying to understand why a trial has produced a particular result.

### Challenges of VPA and Public Understandings of Trial Methodologies

While the VPA method had several advantages in exploring contextualized interpretations of the data and some of the mechanisms behind the success of the PLA intervention, a challenge of the method was participants’ misunderstanding of how trial results could contradict their own experiences and observations of behavior change at a community level. When the results did not confirm what participants had observed or experienced, they rejected the validity of the results, the data collection that was completed, or the accuracy of information provided by survey respondents.

Public understandings of scientific concepts, such as trial methodologies, are often based on common sense knowledge as well as the interpretation of information through the lens of a particular culture or society (Bauer, 2009). Engaging participants in discussions about science, including evaluation methodology, therefore presents both opportunities and challenges. It is challenging in terms of potentially feeding into broader discourses of research abuse and scientific misinformation, which may be particularly concerning in settings or with populations that have previous experienced research misuse (Noah, 2003; Stevens & Pletsch, 2002). However, if done effectively, explaining scientific principles to lay publics can also increase the potential for citizen engagement and wider participation in addressing the health issues that affect individual’s everyday lives (Sinatra & Hofer, 2016). The challenge therefore becomes how to effectively explain complex scientific principles, such as the experimental methods on which trials are based, to lay publics. Effective communication about this would need to explain the ways in which data are aggregated as part of population-level surveys, develop understandings of the value of this approach, and explain how this may not correspond with actual experience of individuals or communities. We have not found the solution to this in our use of VPA, but raise it as a challenge arising from the method.

### Contribution to the Field of Mixed Methods

Our findings contribute to the mixed methods literature more broadly by highlighting a potentially significant advantage of using participatory research approaches alongside trials. To a certain extent, the use of VPA produced findings that may be similar to process evaluations, including contextual factors that may have had an impact on the intervention, or the causal mechanisms of an intervention (Moore et al., 2015). The difference being that VPA allows for the collection of data that support deeper understandings of an intervention’s causal mechanisms after the analysis of trial results. We found a high degree of triangulation between the VPA results and the process evaluation analysis conducted as part of the DMagic trial. As presented in the findings, the use of VPA in the DMagic trial identified participants’ own understanding that if they were effectively preventing diabetes through changing their behaviors then they would not need to go to the doctor or other health care provider. It also highlighted factors that brought about changes in individual behavior such as the use of visual tools, participatory group discussion, and the role of informal conversation in disseminating knowledge to a wider audience in the community. In the process evaluation research, participants similarly discussed the development of knowledge about diabetes prevention and control through the discursive participatory format of the group. This format enabled them to become confident in that knowledge in order to share it with others, which enabled behavior change in the wider community. The group also strengthened their social network, which enabled them to collectively interact with others to change behaviors. Both the VPA and process evaluation research showed that the groups addressed social norms, creating an enabling social context within the household and community to enact healthy behaviors (Morrison et al., 2019).

### Using Participatory Methods as Part of Trials

The success of VPA as a method highlights the need for improvements in the use of participatory methods as part of mixed methods research. While the potential benefits of increasing the involvement of participants in the research process is increasingly well recognized as a means of addressing complex health problems, the majority of interventions undergoing RCTs aim to accomplish this through patient or participant consultation (Davis et al., 2019). This is a far cry from the form of meaningful engagement described by advocates of participatory research (Cornwall & Jewkes, 1995). Moreover, while the benefits of participatory methods have been widely established with and by qualitative researchers (Chambers, 1994; Lewin, 1946; Minkler & Wallerstein, 2008), the potential for participants to be actively involved in trial-related research activities remains largely unexplored.

## Conclusion

In conclusion, the VPA method provides a feasible and potentially valuable approach to increasing the utilization of participatory methods as part of trials and benefiting from the rich insights that this type of meaningful engagement can offer. The added value of the VPA method is that it can elicit this type of information after a trial has been completed in ways that can complement or further examine the hypotheses generated by process evaluations while the trial is underway. In this way, VPA should not be seen as a replacement for high-quality process evaluations, or the much-needed use of qualitative data to inform interventions before a trial begins. Rather, it provides an additional tool for building a mixed methods approach into the final analysis phase of RCTs and further exploring the “black box” of why and how complex health interventions have worked (or did not work) in different settings.
